# Post-Marketing Safety Surveillance of the Salvia Miltiorrhiza Depside Salt for Infusion: A Real World Study

**DOI:** 10.1371/journal.pone.0170182

**Published:** 2017-01-26

**Authors:** Ying-Ying Yan, Yi-Heng Yang, Wei-Wei Wang, Yu-Ting Pan, Si-Yan Zhan, Ming-Yang Sun, Hong Zhang, Suo-Di Zhai

**Affiliations:** 1 Department of Pharmacy, Peking University Third Hospital, 49 North Garden Rd., Haidian District Beijing, China; 2 School of Public Health, Peking University Health Center, 38 Xueyuan Rd., Haidian District, Beijing, China; 3 Department of Pharmacy Administration and Clinical Pharmacy, School of Pharmaceutical Sciences, Peking University Health Center, 38 Xueyuan Rd., Haidian District, Beijing, China; 4 State Administration of Traditional Chinese Medicine of the People’s Republic of China, 55 Xingfu Yicun, Chaoyang District, Beijing, China; National Chiao Tung University College of Biological Science and Technology, TAIWAN

## Abstract

**Background:**

Salvia Miltiorrhiza Depside Salt for Infusion (SMDS) is made of a group of highly purified listed drugs. However, its safety data is still reported limitedly. Compared with the clinical trials, its safety in the real world setting is barely assessed.

**Objective:**

To investigate the safety issues, including adverse events (AEs), adverse events related to SMDS (ADEs), and adverse drug reactions (ADRs) of the SMDS in the real world clinical practice.

**Methods:**

This is a prospective, multicenter, pharmacist-led, cohort study in the real world setting. Consecutive patients prescribed with SMDS were all included in 36 sites. Pharmacists were well trained to standardized collect the patients information, including demographics, medical history, prescribing patterns of SMDS, combined medications, adverse events, laboratory investigations, outcomes of the treatment when discharge, and interventions by pharmacists. Adverse events and adverse drug reactions were collected in details. Multivariate possion regression analysis was applied to identify risk factors associated with ADEs using the significance level (α) 0.05. ClinicalTrials.gov Identifier: NCT01872520.

**Results:**

Thirty six hospitals were participated in the study and 30180 consecutive inpatients were included. The median age was 62 (interquartile range [IQR], 50–73) years, and male was 17384 (57.60%) among the 30180 patients. The incidences of the AEs, ADEs and ADRs were 6.40%, 1.57% and 0.79%, respectively. There were 9 kinds of new ADEs which were not on the approved label found in the present study. According to the multivariate analysis, male (RR = 1.381, P = 0.009, 95%CI [1.085~1.759]), more concomitant medications (RR = 1.049, P<0.001, 95%CI [1.041~1.057]), longer duration of SMDS therapy (RR = 1.027, P<0.001, 95%CI [1.013~1.041]), higher drug concentration (RR = 1.003, P = 0.014, 95%CI [1.001~1.006]), and resolvent unapproved (RR = 1.900, P = 0.002, 95%CI [1.260~2.866]) were the independent risk factors of the ADEs. Moreover, following the approved indication (RR = 0.655, P<0.001, 95%CI [0.532~0.807]) was associated with lower incidence of ADEs.

**Conclusions:**

SMDS was well tolerated in the general population. The incidences of the AEs, ADEs and ADRs were 6.40%, 1.57% and 0.79%, respectively. Several risk factors of its ADEs have been identified. It is recommended to follow the instructions when prescribing and administrating SMDS in the real world clinical practice.

## Introduction

Traditional Chinese Medicine (TCM) injections are produced from herbals based on the theory of TCM using modern techniques and methods. However, since severe adverse drug reactions (ADRs) were identified in 2004, including anaphylactic shock and lethal anaphylaxis, TCM injections were highlighted in decades because of its safety issues in China[1–3]. The extraction progresses from the herbals and the purity of the injections have been considered as the most important causes for the severe ADRs[1,2].

DanShen (*Salvia miltiorrhiza*) is a traditional Chinese herb which is widely used in the treatment of angina pectoris, hyperlipidemia, and acute ischemic stroke for thousands of years[4]. Made of highly purified listed drugs, Salvia Miltiorrhiza Depside Salt for Infusion (SMDS, the water-soluble purified compounds from DanShen) was approved by China Food and Drug Administration in 2005 as a TCM injection which contains magnesium salvianolate B (≥85%), rosmarinic acid (≥10.1%), lithospermic acid (≥1.9%) and other 5 homologs[5–7]. Including 7 phenolic hydroxyl groups, magnesium salvianolate B is one of new generation of natural antioxidants[8]. As free radical plays an important role in normal physiological functions and human disease, the antioxidant stress is the vital part of progress in protecting ischemic myocardium[8]. Previous cardiovascular pharmacology studies showed that magnesium salvianolate B have pleiotropic effects, including antioxidant effect, antiplatelet aggregation and antithrombotic effect. It can also promote cardiac angiogenesis, protect myocardial cells from apoptosis, inhibit ischemia and hypoxia of myocardial injury, protect endothelial cell ion and etc. [5] The efficacy of SMDS has been proved in its previous clinical trials[9–11]. The indication was to treat coronary heart disease (CHD), mainly for stable angina.

SMDS is one of the largest prescription drugs used in the treatment of cardiovascular disease in China. Different from other TCM injections which may contain multiple unknown compounds, the components of SMDS were clarified which may lead to less severe anaphylaxis. However, there is lacking of evidence to support these hypotheses. In its previous clinical trials, a few ADRs have been reported and were all mild to moderate[10,11]. With more and more widely use in the general population, safety issues which were not found in the standard world might begin to be identified. Compared with the patients who were screened by strictly inclusion and exclusion criteria in the clinical trials, general population, especially the special populations who might have more risk factors when using SMDS, could be included in the real world studies. Few studies reported the safety of SMDS among these patients.

Considering the incidence, manifestations, outcomes and risk factors of SMDS’ ADEs in the real world setting are still unknown, we designed and organized a prospective, multicenter, pharmacist-led, cohort study to investigate the safety issues and the prescribing patterns of SMDS in real world clinical practice.

## Methods

This was a prospective, multicenter, pharmacist-led, cohort study. Using ideals of “real world study” [12], we collected consecutive patients who prescribed and administrated SMDS using surveillance method in previously selected hospitals. This observational study was designed and conducted by pharmacists.

### Ethics

This study was approved by Peking University Third Hospital Medical Ethics Committee with waiver informed consent (reference number: IRB00006761-2011093). The reasons that we applied for waiver are as follows. (1) Our study is an observational, non-interventional study. Only when there is an irrational drug been used will pharmacists conduct an intervention. Our study involves no more than minimal risk to the patients. The only risk is patients’ privacy which will be well protected and maintain confidential during the study. (2) The waiver will not adversely affect the rights and the welfare of the patients. (3) Without the waiver, a registration bias will be brought by the informed consent and the prescribing behavior by physicians will be influenced[13]. In our study, 35 collaborating hospitals accepted central ethics except for Huashan Hospital which obtained local approval by Fudan University Huashan Hospital Ethics Committee.

### Site selection and inclusion criteria

According to the sales of SMDS in different regions and hospitals, as well as the hospital levels and types, we invited department of pharmacy in excellent scientific research ability into our research. The list of hospitals could represent the prescribing distribution of SMDS in mainland China. Hospital list is displayed in S1 Appendix, including tertiary hospitals and secondary hospitals, general hospitals, specialized hospitals, and traditional Chinese medicine hospitals.

Study sites were required to enroll patients consecutive. Patients who were prescribed SMDS (Shanghai Green Valley Pharmaceutical Co, Ltd) during APR 2012 to JAN 2015, including inpatients, patients in the emergency, and outpatients with safety information, were all included in each site. Considering the t1/2 of SMDS is only 2.87h, patients were followed up until discharged or 14 days after SMDS discontinued [9].

### Sample size

PASS 11.0.7 was used to calculate the sample size. Considering the incidence of adverse drug reaction (0.56%) in the Phase IV study, we hope that we could observe the incidence of ADR less than 0.1%. To achieve a precision of 0.5% with α of 0.05, we need to sample 25334 patients. Assuming 20% of the sample lost to follow-up, we need to sample 30400 patients in this study.

### Data collection

**Fig 1:**
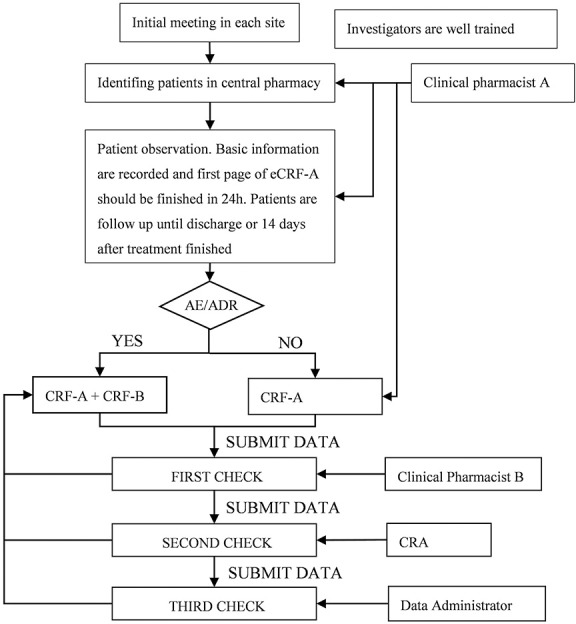
Flow chart of data collection.

Standardized case report forms (CRF) and standardized data collection procedure were conducted before the study started. We trained investigators who were mainly clinical pharmacists with details of the protocol, data elements, data dictionary, and web based electronic data system. Data are all collected and submitted in a web based electronic data system (http://210.14.78.123:88/portal/root/gcp_data_dsdf/index.jsp) which was secure and password-protected by the local investigators in each site. The flow chart of data collection was displayed in Fig 1. Clinical pharmacists identified patients who met the inclusion in central pharmacy every day, then recorded patients’ information and observed adverse events. Patients’ outcomes would be measured by physicians and then recorded by pharmacists.

### Data elements

The data elements in this study were developed and determined by principle investigators, clinical and pharmacy experts of the scientific committee. Taken objectives and feasibility of the project into consideration, data elements include patient demographics, medical history, diagnoses and risk factors, prescribing pattern of SMDS, combined medication, adverse events, lab results, outcomes when discharge, and interventions by pharmacists. All data elements collected by pharmacists who were well trained before patients enrolled in. Pharmacists were trained with a standardized set containing variables dictionary and definitions. CRF consisted of CRF-A and CRF-B. CRF-A contained all the data elements except for the information of adverse events contained in CRF-B. The variables of medical history and diagnoses were coded as ICD-10, while the variables of adverse events were coded as MedDRA, and the variables of medication were coded as WHO-ATC codes.

### Data management and quality control

Each patient was assigned a unique ID when investigator submitted the basic information. Duplicate records were identified through these variables: hospital, period of treatment of SMDS, and hospital admission ID or outpatient ID. Data submitted were checked 3 times each patient. Three times check were conducted by second pharmacist, CRA, and data administrator independently and separately. If there was any problem, CRFs will be returned to the first pharmacist only who could input and revise the data. Data input tracking, regular alerts, and queries from CRA were used to support the accuracy completion of eCRFs.

### Adverse events recorded and interpreted

**Fig 2:**
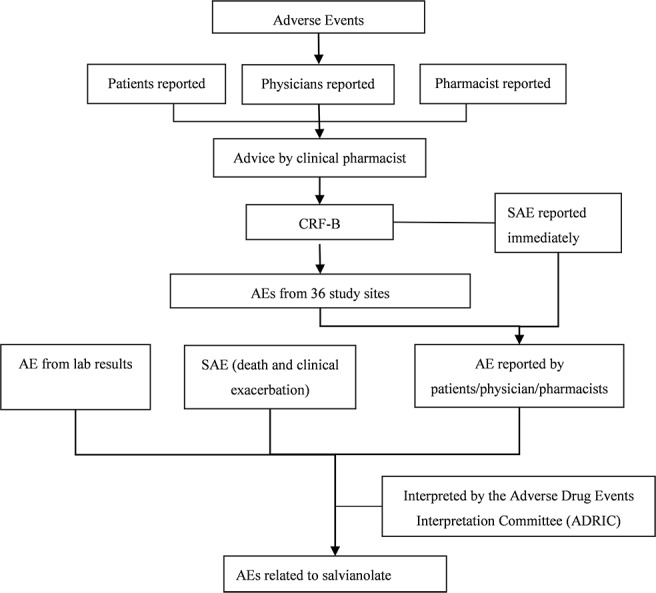
Flow chart in identifying ADRs/AEs by pharmacists.

All adverse events related to SMDS were recorded by clinical pharmacists. The flow chart was displayed in Fig 2. Adverse events (AEs) were reported from 4 types of sources: the death, the exacerbation of disease when patient discharge, pharmacists reported and abnormal lab results. According to the phase III and IV trials, we focused on these laboratory investigations which might be signals of AEs as follows: renal function test, liver function test, routine blood test, coagulability and hemostatic function test and fecal occult-blood test. In order to explain and interpret the ADR by the Adverse Drug Events Interpretation Committee (ADEIC), medical records were collected in patients who experienced an adverse event. AEs were categorized as certain, probable/likely, possible, unlikely, conditional/unclassified or unassessable/unclassifiable by WHO-UMC causality assessment system (S2 Appendix)[14,15]. Levels beyond possible were defined as adverse events related to SMDS (ADEs). ADEs were categorized as mild, moderate and severe (S3 Appendix)[16]. According to the definition of adverse drug reaction (ADRs) of WHO, we checked the prescribing pattern of the SMDS of patients who experienced ADEs, and only ADEs with on label use were defined as ADRs.

### Statistics analysis

Statistics analysis was performed using SAS 9.2. Categorical data was presented as frequencies and compared by chi-square and Fisher exact tests as appropriate. Continuous data was presented as the mean ± SD and median (interquartile range [IQR]) as appropriate, and compared by factorial analysis of variance. Univariate poisson regression was firstly used to identify the potential predictor. Multivariate poisson regression analysis was used to identify statistically significant associated risk factors by significance level (α) 0.05, and obtain the relative risk (RR) and 95% confidence intervals (CIs). All the available patients were included in the univariate and multivariate poisson regressions.

## Results

### Basic characteristics of the included patients

Thirty six hospitals participated in the study and 30180 consecutive inpatients were included. Considering salvanolate is a TCM injection, Traditional Chinese Medicine hospitals were also included (30.56%). Male was 17384 (57.60%) among the 30180 patients. The median age was 62 (interquartile range [IQR], 50–73), and 44.33% patients were more than 65 years. Drug allergy history was in 3988 (13.21%) patients. The median length of stay was 11 days. Demographic characteristics and patients’ main outcomes during hospital were shown in Table 1.

**Table 1:** Basic characteristic of the included patients.

Variables		Inpatients (n = 30180)
**Hospital type n (%)**	General hospital	23 (63.89%)
	Traditional chinese medicine hospital	11 (30.56%)
	Specialized hospital	2 (5.5 6%)
**Demographic characteristics**	Gender, male n (%)	17384 (57.60%)
	Age (median, Q1, Q3)	62 (50, 73)
	Ethnic Han n (%)	26922 (89.20%)
	Smoking n (%)	6990 (23.16%)
	Alcohol drinking n (%)	4521 (14.97%)
	Drug allergy history n (%)	3988 (13.21%)
	Food allergy history n (%)	182 (0.60%)
**Length of stay**		11.00 (8.00,16.00)
**Death in hospital n (%)**		201 (0.67%)

The usages of SMDS in real world clinical setting were described in Table 2. According to the drug package inserts, patients diagnosed as coronary heart disease (CHD) were 42.53% and three quarters patients administrated SMDS followed the instruction dosage requirement (200mg per day). The mainly off label use information in prescription patterns of SMDS were shown in Table 2. The median therapy period is 7 days. Top 5 combination therapies were all attached to cardiovascular medications, including aspirin, clopidogrel, isosorbide mononitrate, metoprolol, and atorvastatin.

**Table 2:** The usage of SMDS in real world clinical setting.

Variables		Inpatients (n = 30180)
**Prescription patterns of SMDS**	Coronary heart disease n (%)	12836 (42.53%)
	Angina(including stable and unstable) n (%)	6285 (20.83%)
	Injury, poisoning and certain other consequences of external causes n (%)	1081 (3.58%)
	Diseases of the circulatory system, except for CHD n (%)	976 (3.23%)
	Dose of SMDS 200mg/d n (%)	22409 (74.25%)
	More than 200mg/d n (%)	1558 (5.16%)
	Less than 200mg/d n (%)	6213 (20.59%)
	Preparation concentration of SMDS (median, Q1, Q3)	0.80 (0.80, 0.80)
	Therapy period (median, Q1, Q3)	7.00 (4.00, 11.00)
**Concomitant medications**	Aspirin n (%)	12624 (41.83%)
	Clopidogrel n (%)	8218 (27.23%)
	Isosorbide Mononitrate n (%)	7019 (23.26%)
	Metoprolol n (%)	6728 (22.29%)
	Atorvastatin n (%)	6480 (21.47%)

### The incidence and the casualty assessment of AEs, ADEs and ADRs

AEs were identified from 4 routes in this study: death during therapy, exacerbation of the origin disease, reported by pharmacists and abnormal laboratories tests. According to the WHO-UMC causality categories, AEs which levels beyond possible were considered as ADEs (Table 3). Among 30180 patients, 1933 AEs were identified in 1678 patients. The incidence of the AEs was 6.40%. After interpreted by ADEIC, 473 AEs were classified as ADEs (1.57%) in 440 patients (1.46%). Among the 473 ADEs, 237 ADEs were identified as ADRs (0.79%) in 224 patients (0.74%) (Table 4). As shown in Table 5, AEs were ranged from 0.00% to 11.77%, ADEs were 0.00% to 6.21%, and ADRs were 0.00% to 2.40% in 36 hospitals, relatively.

**Table 3:** Casualty assessments of the AEs.

**Casualty assessment**	**Original disease exacerbation**	**Death during therapy**	**Reported by pharmacists**	**Laboratories tests**	**Total**
**Certain**	0	0	0	0	0
**Probable**	0	0	3	16	19
**Possible**	0	0	6	448	454
**Unlikely**	17	162	9	561	749
**Conditional**	0	1	0	0	1
**Unassessable**	4	16	0	690	710
**Total**	21	179	18	1715	1933

**Table 4:** AEs, ADEs and ADRs identified by 4 routes.

**Sources of AEs**		**AEs**	**ADEs**	**ADRs**
**Death during therapy**		179 (0.59%)	0 (0.00%)	0 (0.00%)
**Accelerate of the disease**		21 (0.07%)	0 (0.00%)	0 (0.00%)
**Reported by pharmacists**		18 (0.06%)	9 (0.03%)	2 (0.01%)
**Laboratory tests**	Renal dysfunction	541 (1.79%)	7 (0.02%)	5 (0.02%)
	Liver dysfunction	344 (1.14%)	228 (0.76%)	121 (0.40%)
	Positive fecal occult blood	282 (0.93%)	26 (0.09%)	17 (0.06%)
	Abnormal of INR	276 (0.91%)	64 (0.21%)	29 (0.10%)
	thrombocytopenia	272 (0.90%)	139 (0.46%)	63 (0.21%)
**Total**		1933 (6.40%)	473 (1.57%)	237 (0.79%)

**Table 5:** AEs, ADEs and ADRs and general information in 36 hospitals.

**Hospitals**	**Number of included patients**	**AEs (%)**	**ADEs (%)**	**ADRs (%)**
**Peking University Third Hospital**	318	31 (9.75%)	3 (0.94%)	0 (0.00%)
**The General Hospital of the People’s Liberation Army**	2000	156 (7.80%)	75 (3.75%)	48 (2.40%)
**The Military General Hospital of Beijing PLA**	1000	139 (13.90%)	27 (2.70%)	20 (2.00%)
**Xijing Hospital**	2998	164 (5.47%)	34 (1.13%)	21 (0.70%)
**Tangdu Hospital**	1007	109 (10.82%)	38 (3.77%)	20 (1.99%)
**Shaanxi Province Hospital of Traditional Chinese Medicine**	715	5 (0.70%)	2 (0.28%)	0 (0.00%)
**Shaanxi Provincial People’s Hospital**	495	48 (9.70%)	4 (0.81%)	1 (0.20%)
**Yanan University Affiliated Hospital**	604	40 (6.62%)	11 (1.82%)	3 (0.50%)
**The First Affiliated Hospital of Xinjiang Medical University**	2999	77 (2.57%)	23 (0.77%)	9 (0.30%)
**People’s Hospital of Xinjiang Uygur Autonomous Region**	1208	75 (6.21%)	18 (1.49%)	13 (1.08%)
**Traditional Chinese Medicine Hospital of Xinjiang Uygur Autonomous Region**	1452	105 (7.23%)	34 (2.34%)	26 (1.79%)
**The Fifth Affiliated Hospital of Xinjiang Medical University**	600	25 (4.17%)	3 (0.50%)	1 (0.17%)
**Chest Hospital of Xinjiang Uygur Autonomous Region**	503	48 (9.54%)	14 (2.78%)	8 (1.59%)
**The First Affiliated Hospital of the Medical College, Shihezi University**	1862	75 (4.03%)	14 (0.75%)	5 (0.27%)
**Wuhan Asia Heart Hospital**	1394	49 (3.52%)	27 (1.94%)	14 (1.00%)
**Hubei Provincial Hospital of TCM**	1013	29 (2.86%)	2 (0.20%)	0 (0.00%)
**Wuhan Integrated TCM and Western Medicine Hospital**	301	5 (1.66%)	1 (0.33%)	1 (0.33%)
**Wuhan Hospital of Traditional Chinese Medicine**	301	18 (5.98%)	2 (0.66%)	0 (0.00%)
**The Second Hospital of Hebei Medical University**	2217	261 (11.77%)	35 (1.58%)	10 (0.45%)
**Weinan Central Hospital**	299	19 (6.35%)	8 (2.68%)	0 (0.00%)
**Hebei General Hospital**	1500	77 (5.13%)	27 (1.80%)	17 (1.13%)
**Guang’anmen Hospital Affiliated to China Academy of Chinese Medical Sciences**	299	41 (13.71%)	6 (2.01%)	2 (0.67%)
**Longhua Hospital Shanghai University of TCM**	306	59 (19.28%)	19 (6.21%)	2 (0.65%)
**Huashan Hospital, Fudan University**	351	52 (14.81%)	13 (3.70%)	7 (1.99%)
**Shuguang Hospital Shanghai University of Traditional Chinese Medicine**	302	49 (16.23%)	9 (2.98%)	2 (0.66%)
**The First Affiliated Hospital of Bengbu Medical College**	1458	42 (2.88%)	3 (0.21%)	1 (0.07%)
**First Hospital of Qinhuangdao**	302	12 (3.97%)	4 (1.32%)	1 (0.33%)
**Hospital of Chengdu Office of People’s Government of Tibetan Autonomous Region (Hospital.C.T.)**	299	15 (5.02%)	1 (0.33%)	0 (0.00%)
**Sichuan 2^nd^ Hospital of TCM**	201	15 (7.46%)	6 (2.99%)	0 (0.00%)
**Zhangjiagang Hospital of Traditional Chinese Medicine**	297	25 (8.42%)	2 (0.67%)	2 (0.67%)
**Minhang District Central Hospital**	305	8 (2.62%)	0 (0.00%)	0 (0.00%)
**AVIC 363 Hospital**	307	3 (0.98%)	0 (0.00%)	0 (0.00%)
**Shanghai Huangpu District Central Hospital**	300	0 (0.00%)	0 (0.00%)	0 (0.00%)
**Central Hospital of Qingpu District, Shanghai**	210	10 (4.76%)	3 (1.43%)	2 (0.95%)
**Affiliated Hospital Of Chengdu University**	304	47 (15.46%)	5 (1.64%)	1 (0.33%)
**China S.C.H.J. Hospital of TCM**	153	0 (0.00%)	0 (0.00%)	0 (0.00%)
**Total**	30180	1933	473	237

### The manifestations of ADEs

There were 9 kinds of new ADEs which were not in the approved instruction found in the present study, including rash, pruritus, erythemas, palpitations, fecal occult blood positive, international normalised ratio increased, blood bilirubin increased, blood creatinine increased and platelet count abnormal. With more details in ADEs reported by pharmacists’ routes, the severity, disposition and recovery data of ADEs in this route were shown in Table 6. Most of these ADEs were all symptomatic and mild to moderate. After SMDS withdrawal and some cases with symptomatic treatment, all patients took turn for better. Most of ADEs from abnormal laboratory tests were mild to moderate. The severity and disposition of the abnormal laboratory tests were shown in Table 7. All severe ADEs were platelet count abnormal. However, the disposition and recovery data were limited because these data were mining from database.

**Table 6:** ADEs reported by pharmacists.

**Systems**	**Manifestations**	**Severity (n)**	**Disposition**	**Recovery**
**Skin and subcutaneous tissue disorders**	Rash	Mild (2)	Withdrawal; deal with symptoms: epinastine, diphenhydramine,	Improvement (1); Cured (1)
	Pruritus	Mild (1)	Withdrawal; deal with symptoms: loratadine	Cured (1)
	Erythemas	Mild (1)	Withdrawal	Improvement (1)
**Nervous system disorders**	Headache	Mild (1)	Withdrawal	Improvement (1)
	Dizziness	Mild (1)	Withdrawal	Improvement (1)
**Cardiac disorders**	Palpitations	Mild (1)	Withdrawal	Improvement (1)
**Investigations**	Transaminases increased	Mild (1)—moderate (1)	Withdrawal; deal with symptoms: glutathione; sodium glucuronate; bicyclol; polyene phosphatidylcholine; vitamin C	Improvement (2)

**Table 7:** ADEs identified from abnormal laboratory tests.

**Systems**	**Manifestations**	**Severity (n)**	**Disposition**
**Investigations**	Transaminases increased	Mild (211)—moderate (8)	Polyene phosphatidylcholine, glutathione, diammonium glycyrrhizinate, glycyrrhizin, magnesium isoglycyrrhizinate, silibinin, ursodeoxycholic acid, ademetionine 1,4-butanedisulfonate
	Blood bilirubin increased	Mild (7)—moderate (2)	
**Investigations**	Blood creatine increased	Mild (7)	Compound α-ketoacid
**Investigations**	Platelet count abnormal	Mild (90)—moderate (33)—severe (16)	-
**Investigations**	Fecal occult blood positive	Mild (26)	-
**Investigations**	International normalised ratio increased	Mild (63)—moderate (1)	-

### Risk factors of ADEs by poisson regression

According to univariate analysis, higher incidence of ADEs was associated with male(RR = 1.460, P = 0.003, 95%CI [1.137~1.875]), smoking (RR = 1.544, P<0.001, 95%CI [1.260~1.892]), alcohol drinking (RR = 1.519, P<0.001, 95%CI [1.207~1.912]), higher single dose (RR = 1.003, P = 0.002, 95%CI [1.001~1.004]), longer duration of therapy (RR = 1.051, P<0.001, 95%CI [1.039~1.063]), higher concentration (RR = 1.004, P<0.001, 95%CI [1.003~1.006]), higher infusion rate (RR = 1.017, P = 0.032, 95%CI [1.001~1.032]), resolvent unapproved (RR = 1.805, P = 0.002, 95%CI [1.252~2.601]) and more concomitant medications (RR = 1.057, P<0.001, 95%CI [1.050~1.063]).

According to multivariate analysis, male (P = 0.009), more concomitant medications (P<0.001), longer duration of SMDS therapy (P<0.001), higher drug concentration (P = 0.014), and resolvent unapproved (P = 0.002) were independent risk factors of the ADEs. Moreover, following the approved indication (P<0.001) was associated with lower incidence of ADEs (Table 8).

**Table 8:** Risk factors of ADEs by multivariable poisson regression.

**Risk Factors**	**RR**	***P***	**95% Confidence Interval**
**Male**	1.381	0.009^a^	1.085~1.759
**Smoking**	1.157	0.327	0.865~1.547
**Alcohol drinking**	1.146	0.382	0.844~1.557
**Bleeding history**	1.370	0.304	0.751~2.499
**Infusion rate**	1.016	0.163	0.994~1.038
**Numbers of concomitant medication**	1.049	<0.001^a^	1.041~1.057
**Dose of SMDS**	0.998	0.164	0.995~1.001
**Duration of SMDS therapy**	1.027	<0.001^a^	1.013~1.041
**Drug concentration**	1.003	0.014^a^	1.001~1.006
**Resolvent unapproved**	1.900	0.002^a^	1.260~2.866
**Indication**	0.655	<0.001^a^	0.532~0.807

^a^P = 0.05.

## Discussion

In this prospective, multicenter, pharmacist-led, cohort study in the real world setting, we described the patients’ characteristics when using SMDS, identified the incidences and manifestations of AEs, ADEs, and ADRs of SMDS, and also identified the risk factors of its ADEs.

There were 4 routes to identify the AEs in this study, including death during the therapy, exacerbation of the origin disease, reported from pharmacists, and laboratories investigation. Compared with other post-marketing safety surveillance of TCM injection which source of AEs was only from pharmacists reported[17], data were fully used to identify and describe AEs in our study. With details of medical records of AEs in 3 routes (reports from the death, exacerbation of the origin disease and the reports from pharmacists), the severity, disposition and recovery of the AEs could be clarified. However, the real world setting led to the loss of disposition and recovery data in the laboratory investigation route.

There was an overestimate or underestimate of the incidence of the AEs. All the AEs/ADRs were determined by center evaluation, and the suspected AEs were all determined as AEs which might lead to overestimate. The factors that might cause underestimate were as follows. Firstly, although under reporting might still remain in this present study, its rate may be significant lower than SRS. Moreover, we focused on the most important laboratories investigations based on the results of SMDS phase III and IV clinical trials which might lead to fewer kinds of lab tests. In addition, the frequency of the laboratories investigations in the real word setting was less than which in randomized controlled trials. These were all risks of the underestimate of the incidence of the AEs.

Hospital pharmacists have been invited to submit the ADR reports in China since the China adverse drug reaction monitoring system was established in 1989[18]. Pharmacists are now making a considerable contribution to the SRS in China[19]. Su et al reported that although they need more training and education, hospital pharmacists in the northern region of China had a reasonable knowledge and positive attitudes towards pharmacovigilance [20]. In 1982, Borden et al reported the feasibility to establish a registry by pharmacists when medication was dispensed [21]. Moreover, selection bias from 2 aspects would be avoided in drug safety study led by pharmacists. Firstly, without pharmacy data from the whole hospital, physicians might only focus on their own specialties which led to miss patients meeting the eligibility criteria. Then, there might be selective prescribing behavior by physicians in the observational study [22]. For example, physicians might not prescribe the research medication to patients who developed serious comorbid conditions [22]. In addition, as the perspective of pharmacists’ role has changed a lot both from dispensing to clinical practice and from focusing on drugs to patients in decades in China, this study also could present pharmacists an opportunity to provide clinical services to the patients and physicians.

SMDS has been widely used in real world clinical practice since it was approved by CFDA in 2005. It is in an urgent need to focus on the safety of the off label use in different real world clinical settings. Some previous cell-level studies and animal studies indicated that the main component of SMDS (magnesium salvianolate B) may have multiple effects in ameliorating bone healing [23], neuroprotective potentials [24], hepatocytes protection [25], lung injury [26], renal protection [27,28],etc. However, there is no study to evaluating its effectiveness and safety in these areas in human as well as the cost. Future studies are needed to investigate the effectiveness and safety of SMDS off-label use.

Database has been used to evaluate the drug safety in recent years. However, the available databases are difficult to be accessed, shared and linked in China. Without an appropriate mechanism to use, manage and link the database, single database, either one hospital medical records or part of medical insurance records, becomes an information silo. In this condition, many registries with large sample sizes were established and our study is one of which meet the demands of the drug safety evaluation.

There are several other strengths in this study. One is the representative of the sample. This pharmacist-led and large sample size design could avoid the selection bias maximum likelihood. Although the hospital is conducted mainly by physician, Wuhan Asia Heart Hospital is a cardiology specialized hospital which only has 2 clinical departments and could follow the protocol. In addition, we selected diverse hospital representatives in different levels and types. Secondly, this cohort study is similar to products registry. With comprehensive data elements and data collection, the clinical prescribing pattern of SMDS in real world setting, including rational and irrational drug use, could be firstly assessed, and the risk of different clinical practice could be evaluated. Thirdly, the present study is the first study to illustrate the safety issue and the risk factors of SMDS.

There are also some limitations of this study. Very few outpatients were included in the study because of feasible difficulties. Since the electronic medical record system are quite different among each hospital in mainland China, follow-up information of outpatients was not available that led to many exclusion because of lacking full safety information. Then, it is a challenge to evaluate effectiveness of SMDS with a single arm study design. However, with relatively full information collected, including the combined medications and outcomes of the disease, this study could provide the effectiveness of other medications indirectly when group them differently despite of the bias. Thirdly, the long-term safety is difficult to investigate because of the relatively short follow-up. However, with the comprehensive study on the pharmacokinetics when compared with other TCM injection, adverse events may explained by its PK profile[7,9,29]. Considering the pharmacokinetics parameters (t1/2 of SMDS is 2.87h; t1/2 of the metabolites are all 0.49–0.63h[9]; SMDS could be rapidly metabolized; and SMDS excreted into bile rapidly mostly as methylated metabolites), our follow up duration could cover the progress of the metabolism and excretion to some extent.

## Conclusions

In this prospective, multicenter, pharmacist-led, post marketing, cohort study, SMDS were well tolerated in the general population. The incidences of the AEs, ADEs and ADRs were 6.40%, 1.57% and 0.79%, respectively. Several risk factors of ADEs have been identified in the real world setting. It is recommended to follow the instructions when prescribing and administrating SMDS in the real world clinical practice. Future studies should be considered to investigate the effectiveness and safety of the off-label use.

## Supporting Information

S1 Appendix Hospitals participated in the study. (DOCX)

S2 Appendix Causality assessment of AEs. (DOCX)

S3 Appendix Severity of suspected ADRs. (DOCX)

S1 Table STROBE Statement—Checklist of this cohort study. (DOC)
