# Supplementary material for: Post-Marketing Safety Surveillance of the Salvia Miltiorrhiza Depside Salt for Infusion: A Real World Study
Source: PLoS One. 2017 Jan 26;12(1):e0170182. doi: 10.1371/journal.pone.0170182 (PMC5268476; doi:10.1371/journal.pone.0170182)
Supplement: S1 Appendix — (DOCX) [file pone.0170182.s001.docx]

**S1 Appendix. Hospitals participated in the study.**

| Hospitals | | Province | | City | | Hospital Class | | Type | |
| --- | --- | --- | --- | --- | --- | --- | --- | --- | --- |
| Peking University Third Hospital | | Beijing | | Beijing | | Tertiary Hospital | | General Hospital | |
| The General Hospital of the People's Liberation Army | | Beijing | | Beijing | | Tertiary Hospital | | General Hospital | |
| The Military General Hospital of Beijing PLA | | Beijing | | Beijing | | Tertiary Hospital | | General Hospital | |
| Xijing Hospital | | Shaanxi | | Xi'an | | Tertiary Hospital | | General Hospital | |
| Tangdu Hospital | | Shaanxi | | Xi'an | | Tertiary Hospital | | General Hospital | |
| Shaanxi Province Hospital of Traditional Chinese Medicine | | Shaanxi | | Xi'an | | Tertiary Hospital | | TCM hospital | |
| Shaanxi Provincial People's Hospital | | Shaanxi | | Xi'an | | Tertiary Hospital | | General Hospital | |
| Yanan University Affiliated Hospital | | Shaanxi | | Yan'an | | Tertiary Hospital | | General Hospital | |
| The First Affiliated Hospital of Xinjiang Medical University | | Xinjiang | | Urumqi | | Tertiary Hospital | | General Hospital | |
| People's Hospital of Xinjiang Uygur Autonomous Region | | Xinjiang | | Urumqi | | Tertiary Hospital | | General Hospital | |
| Traditional Chinese Medicine Hospital of Xinjiang Uygur Autonomous Region | | Xinjiang | | Urumqi | | Tertiary Hospital | | TCM hospital | |
| The Fifth Affiliated Hospital of Xinjiang Medical University | | Xinjiang | | Urumqi | | Tertiary Hospital | | General Hospital | |
| Chest Hospital of Xinjiang Uygur Autonomous Region | | Xinjiang | | Urumqi | | Tertiary Hospital | | Specialized Hospital | |
| The First Affiliated Hospital of the Medical College, Shihezi University | | Xinjiang | | Shihezi | | Tertiary Hospital | | General Hospital | |
| Wuhan Asia Heart Hospital | | Hubei | | Wuhan | | Tertiary Hospital | | Specialized Hospital | |
| Hubei Provincial Hospital of TCM | | Hubei | | Wuhan | | Tertiary Hospital | | TCM hospital | |
| Wuhan Integrated TCM and Western Medicine Hospital | | Hubei | | Wuhan | | Tertiary Hospital | | TCM hospital | |
| Wuhan Hospital of Traditional Chinese Medicine | | Hubei | | Wuhan | | Tertiary Hospital | | TCM hospital | |
| The Second Hospital of Hebei Medical University | | Hebei | | [Shijiazhuang](http://www.baidu.com/link?url=ik4wiGt4MxqLOKIERMJE1oBiCuA9HvO-m_4uElyD1wme3Werycizbza-JfXSbUPCEdCV0W1_YgkEuIUWvg3W2qIPbqTMW2LK_JInLN8E0LpBk_vba8MZJU-si9F0YzeV) | | Tertiary Hospital | | General Hospital | |
| Weinan Central Hospital | | Shaanxi | | Weinan | | Tertiary Hospital | | General Hospital | |
| Hebei General Hospital | | Hebei | | [Shijiazhuang](http://www.baidu.com/link?url=ik4wiGt4MxqLOKIERMJE1oBiCuA9HvO-m_4uElyD1wme3Werycizbza-JfXSbUPCEdCV0W1_YgkEuIUWvg3W2qIPbqTMW2LK_JInLN8E0LpBk_vba8MZJU-si9F0YzeV) | | Tertiary Hospital | | General Hospital | |
| Guang’anmen Hospital Affiliated to China Academy of Chinese Medical Sciences | | Beijing | | Beijing | | Tertiary Hospital | | TCM hospital | |
| Longhua Hospital Shanghai University of TCM | | Shanghai | | Shanghai | | Tertiary Hospital | | TCM hospital | |
| Huashan Hospital, Fudan University | | Shanghai | | Shanghai | | Tertiary Hospital | | General Hospital | |
| Shuguang Hospital Shanghai University of Traditional Chinese Medicine | | Shanghai | | Shanghai | | Tertiary Hospital | | TCM hospital | |
| The First Affiliated Hospital of Bengbu Medical College | | Anhui | | Bengbu | | Tertiary Hospital | | General Hospital | |
| First Hospital of Qinhuangdao | | Hebei | | Qinhuangdao | | Tertiary Hospital | | General Hospital | |
| Hospital of Chengdu Office of People’s Government of Tibetan Autonomous Region (Hospital.C.T.) | | Sichuan | | Chengdu | | Tertiary Hospital | | General Hospital | |
| Sichuan 2nd Hospital of TCM | | Sichuan | | Chengdu | | Tertiary Hospital | | TCM hospital | |
| Zhangjiagang Hospital of Traditional Chinese Medicine | | Jiangsu | | Zhangjiagang | | Tertiary Hospital | | TCM hospital | |
| Minhang District Central Hospital | | Shanghai | | Shanghai | | Secondary Hospital | | General Hospital | |
| AVIC 363 Hospital | | Sichuan | | Chengdu | | Tertiary Hospital | | General Hospital | |
| Shanghai Huangpu District Central Hospital | | Shanghai | | Shanghai | | Secondary Hospital | | General Hospital | |
| Central Hospital of Qingpu District, Shanghai | | Shanghai | | Shanghai | | Tertiary Hospital | | General Hospital | |
| Affiliated Hospital Of Chengdu University | | Sichuan | | Chengdu | | Tertiary Hospital | | General Hospital | |
| China S.C.H.J. Hospital of TCM | | Sichuan | | Hejiang | | Secondary Hospital | | TCM hospital | |
