# Supplementary material for: Post-Marketing Safety Surveillance of the Salvia Miltiorrhiza Depside Salt for Infusion: A Real World Study
Source: PLoS One. 2017 Jan 26;12(1):e0170182. doi: 10.1371/journal.pone.0170182 (PMC5268476; doi:10.1371/journal.pone.0170182)
Supplement: S2 Appendix — (DOCX) [file pone.0170182.s002.docx]

**S2 Appendix. Causality assessment of AEs.**

| Causality term | Assessment criteria |
| --- | --- |
| Certain | - Event or laboratory test abnormality, with plausible time relationship to drug intake - Cannot be explained by disease or other drugs - Response to withdrawal plausible (pharmacologically, pathologically) - Event definitive pharmacologicallyor phenomenologically (i.e. an objective and specific medical disorderor a recognised pharmacological phenomenon) - Rechallenge satisfactory, if necessary |
| Probable / Likely | - Event or laboratory test abnormality, with reasonable time relationship to drug intake - Unlikely to be attributed to disease or other drugs - Response to withdrawal clinically reasonable - Rechallenge not required |
| Possible | - Event or laboratory test abnormality, with reasonable time relationship to drug intake - Could also be explained by disease or other drugs - Information on drug withdrawal may be lacking or unclear |
| Unlikely | - Event or laboratory test abnormality, with a time to drug intake that makes a relationship improbable (but not impossible) - Disease or other drugs provide plausible explanations |
| Conditional / Unclassified | - Event or laboratory test abnormality - More data for proper assessment needed, or - Additional data under examination |
| Unassessable / Unclassifiable | - Report suggesting an adverse reaction - Cannot be judged because information is insufficient or contradictory - Data cannot be supplemented or verified |
