# Supplementary material for: Post-Marketing Safety Surveillance of the Salvia Miltiorrhiza Depside Salt for Infusion: A Real World Study
Source: PLoS One. 2017 Jan 26;12(1):e0170182. doi: 10.1371/journal.pone.0170182 (PMC5268476; doi:10.1371/journal.pone.0170182)
Supplement: S3 Appendix — (DOCX) [file pone.0170182.s003.docx]

**S3 Appendix. Severity of suspected ADRs.**

1. An ADR occurred but no change in treatment with suspected drug.

2. The ADR that required treatment with the suspected drug; will be withheld, discontinued, or otherwise changed. No antidote or other treatment required. No increase in length of stay.

3. The ADR that required treatment with the suspected drug will be withheld, discontinued, or otherwise changed, and/or an antidote or other treatment. No increase in length of stay.

4. Any Level 3 ADR that increases the length of stay by at least one day or the ADR was the reason for admission.

5. Any Level 4 ADR that requires intensive medical care.

6. The ADR caused permanent harm to the patient.

7. The ADR was directly or indirectly linked to death of patient

Level 1 and 2 indicates mild.

Levels 3 and 4 indicate moderate.

Levels 5 and above indicate severe ADRs.
